# Public Spending on Health Service and Policy Research in Canada, the United Kingdom, and the United States: A Modest Proposal

**DOI:** 10.15171/ijhpm.2017.45

**Published:** 2017-04-10

**Authors:** Vidhi Thakkar, Terrence Sullivan

**Affiliations:** Institute of Health Policy Management and Evaluation, University of Toronto, Toronto, ON, Canada.

**Keywords:** Health Policy, Public Spending, Comparative Spending Health Services and Policy Research

## Abstract

Health services and policy research (HSPR) represent a multidisciplinary field which integrates knowledge from health economics, health policy, health technology assessment, epidemiology, political science among other fields, to evaluate decisions in health service delivery. Health service decisions are informed by evidence at the clinical, organizational, and policy level, levels with distinct, managerial drivers. HSPR has an evolving discourse spanning knowledge translation, linkage and exchange between research and decision-maker partners and more recently, implementation science and learning health systems. Local context is important for HSPR and is important in advancing health reform practice. The amounts and configuration of national investment in this field remain important considerations which reflect priority investment areas. The priorities set within this field or research may have greater or lesser effects and promise with respect to modernizing health services in pursuit of better value and better population outcomes. Within Canada an asset map for HSPR was published by the national HSPR research institute. Having estimated publicly-funded research spending in Canada, we sought identify best available comparable estimates from the United States and the United Kingdom. Investments from industry and charitable organizations were not included in these numbers. This commentary explores spending by the United States, Canada, and the United Kingdom on HSPR as a fraction of total public spending on health and the importance of these respective investments in advancing health service performance. Proposals are offered on the merits of common nomenclature and accounting for areas of investigation in pursuit of some comparable way of assessing priority HSPR investments and suggestions for earmarking such investments to total investment in health services spending.

## Evolution of Health Services and Policy Research

Health services and policy research (HSPR) evaluates processes related to the organization, management, delivery, regulation and finance of healthcare services. It is an interdisciplinary research area that combines knowledge from multiple fields: health economics, health policy, health technology assessment, clinical epidemiology and biostatistics, political science, sociology, law, among other fields.^1^ Canada, the United Kingdom and the United States have distinct but overlapping HSPR evolutions. Early work in the United Kingdom in HSPR was rooted in the comparative work of Sir William Petty who compared outcomes in London and Paris hospitals. An excellent historiography of health services in the United States characterizes its origins within the National Institutes of Health* (*NIH).^2^

Following other policy fields, HSPR evolved with a focus on knowledge translation between researchers and decision-makers. This allowed for common ground on evolving priorities with a view to managing priority HSPR research initiatives. Canada, following comparable efforts in the United Kingdom, began a series of ‘listening’ exercises involving researchers, funders and decision-makers in 2001 to set common priorities for HSPR.^3^ A decade later (2013-2014), the Canadian Institute for Health Services and Policy Research (IHSPR) built a topography of national spending on HSPR, following a consultation with funders, decision-makers, researchers and thought leaders.^1^ Canada’s national health research agency (CIHR) groups investments into four spending pillars (biomedical, clinical, health services and policy, and population and public health).^4^ HSPR wins the least amount of public funds among these pillars, despite the promise of HSPR as a key agent of value in health services modernization. Tamblyn et al have noted that in 2010, HSPR was the least funded pillar, having only 6.2% of health research funds at CIHR. This only grew to 7.5% in the year 2015-2016, a modest investment increase.^5^

## Evolution of Health Services and Policy Research in Canada

Canada’s IHSPR^1^ has evolved its program in the last 3 years to align with a more applied, provincially driven, research agenda reflecting reform imperatives. Figure shows the notional evolution of HSPR research in Canada from 1948-2015^4^ with the aspiration of moving from an individual, research contribution model to one which cumulatively informs a learning health system model of continuous self-improvement.^6^ The field matured in Canada with investments from an early national health grant that preceded the Canadian Health Services Research Foundation, IHSPR and the emergence of a national Association for Health Services and Policy Research to advance the field.^7^

**Figure:**
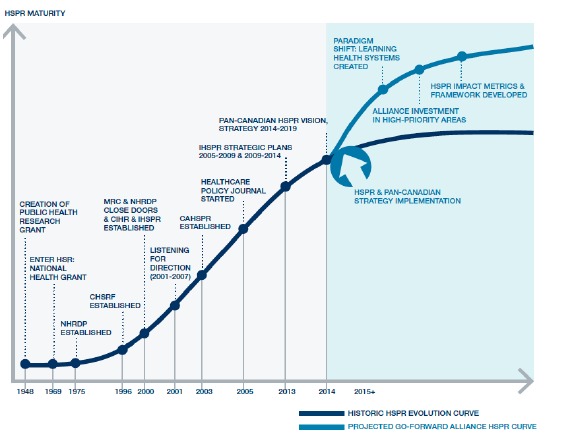
Notional Growth and Evolution of Health Services and Policy Research in Canada With Shift to Learning Health Systems.^4^

In 2014-2016, CIHR’s IHSPR created a pan-Canadian Vision and Strategy for HSPR. The objective of the first phase was to analyse the current state of HSPR investments and account for funding investments in terms of assets, resources, strengths, opportunities and gaps.^4^ IHSPR identified 15 spending categories in its most recent Listening for Direction report. This initiative showed that in the fiscal year of 2010, government only sources invested $131 869 754 million Canadian dollars. This project mapped the flow of HSPR investments by spending categories, geography and recipient institutions.^1^

## Estimating Public Spending in HSPR in the United Kingdom, the United States, and Canada

HSPR research topography is influenced by and in turn influences evolving local policies and delivery systems. Modernizing a health service requires consideration of research funding within a health system.^8^ Estimates of the available public spend on HSPR in Canada, the United States and the United Kingdom were developed to compare respective research investments.^4,9,10^ These estimates do have limitations due to country variation in categorical definitions of HSPR. Publicly available documents were used to calculate the government-only per capita investment on HSPR.

This per capita calculation involved taking the total HSPR research spending by government only for each of the 3 countries standardized to US dollars for the 2010 fiscal year and dividing it by the World Bank population for the same year^ [1] ^. The per capita estimate was verified with an expert from each country for external validity^ [2] ^. The pan-Canadian Vision and Strategy for HSPR validated Canadian spending. Within the United Kingdom, the UK Research and Analysis report was used to estimate spending on HSPR.^9^ For the United States, Academy Health provided federal funding tables developed by Denis and Associates.^10^

## Cross Comparison of HSPR Spending in Canada, the United States and the United Kingdom

As seen in Table in fiscal year 2010, the United States had the greatest per capita investment of US$6.46, representing federal public spending on HSPR. Canada spent much less than the United States, spending US$3.76 per capita. The United Kingdom spent US$2.90 per capita on HSPR.

**Table:** Government Only Spending on HSPR in Canada, the United States, and the United Kingdom

	** Canada Fiscal Year 2010 **	** United States Fiscal Year 2010 **	** United Kingdom Fiscal Year 2010^a^ (est.) **
Government spend on HSPR	US$128 005 970^b^ 34 005 274 people	US$2 000 400 000^c^ 309 346 863 people	US$155 516 835^b^ 62 276 270 people
Per capita public spending on HSPR	US$3.76	US$6.46	US$2.50
Source	World Bank Data + IHSPR Pan-Canadian Vision^4,12^	World Bank data + estimates provided by Dennis & Associates consulted by Academy Health^10,12^	World Bank Data + UK Research and Analysis 2009/2010, first published in 2012^9,12^

Abbreviations: HSPR, Health services and policy research; IHSPR, Canadian Institute for Health Services and Policy Research. a There was no single year of comparison which matched the three countries as the United Kingdom did not report HSPR spending for 2010, but did so for fiscal 2009-2110: (Table 2 in the UK research and analysis 2009-2110 report, which is available at the following link: http://www.ukcrc.org/wp-content/uploads/2014/03/2UKHealthResearchAnalysis-1.pdf). The 2009 estimate of public spending on HSPR was provided by Dr. James Carter, derived from the following link: http://www.hrcsonline.net/sites/default/files/2009_10%20UKHRA%20data%20for%20PUBLICATION_CSV.csv. Growth of government health spending from 2009 to 2010 was a 2% rise in spending (UK Office of National Statistics), which is the health inflation proxy we used to estimate 2010 per-capita HSPR expenditures of 2.96 for the United Kingdom. A 2010 UK-US conversion rate of 1.545 893 was used from Forex Canada (average 2010 rate) and a rate of 0.9707 from Canadian FOREX for the CND to USD conversion. The estimates are certainly conservative estimates for all three countries as we have only taken government funded sources at the national level in the United Kingdom and the United States and federal and provincial level in Canada. b This was obtained from the Pan-Canadian Vision for Strategy and research data tables available at: https://www.cahspr.ca/web/uploads/conference/2015-05-25_Pan_Canadian_Vision_and_Strategy.pdf. c This number was obtained from federal funding tables sent to us by Academy Health from Denis & Associates.

We were unable to identify comprehensive estimates of the United States, state-only investment in HSPR, so the United States is certainly an underestimate of their public HSPR spending. Our estimates do not take into account funding by private sources, charities or municipal sources in the three countries^ [3] ^.

Recent reform within the United States under the Affordable Care Act (ACA) provide a partial context for why their HSPR spending leads the pack.^13^ The ACA has driven large investments in HSPR enabling initiatives including comparative effectiveness studies. Neither Canada nor the United Kingdom have seen comparable HSPR investments.

There is a pressing need to track the impact of increased investments in HSPR on improvements in health system performance and quality. Recent Canadian reforms in CIHR funding have transitioned funds to through the Strategy for Patient Oriented Research Outcomes to more actively support provincial priorities.^14^ Prior to 2010, the United Kingdom had a surge in health service investments under the Blair government which has been associated with improvements in quality over time. One need only look at the performance of the three countries in US Commonwealth Fund surveys and the Organization for Economic Co-operation and Development (OECD) studies to see that the United Kingdom focus on improving health sector performance has shown promise.^15-17^ It remains a challenge to understand, in the absence of better comparative data, why the major US HSPR spending yields relatively mixed results, why Canada fares less well in international comparisons than might be expected relative to its spend on HSPR. Is it possible that the United States and Canada as federated states are more challenged to apply population-based HSPR derived service standards across subnational jurisdictions than it might be in a unitary state like the United Kingdom with one National Health Service (NHS)?

What does appear clear is that high performing institutions appear to be well governed and managed with better quality and performance outcomes.^18^ The challenge is to get better comparative data to understand the impact of HSPR investments and how they interact with intentional managerial and governance processes to improve performance over time within specific organizational, managerial and policy contexts. This will help us understand how HSPR can best be deployed in building capacity for improvement and quality.^19^ Some work is underway through IHSPR in Canada to build better indicators for impact and attribution building on the Canadian Academy of Health Science report on research metrics including return on investment^ [4] ^.

## Improving Comparability: A Modest Proposal

The respective political and economic contexts shed some light on the HSPR spending data in the three countries, with spending on HSPR reflecting the ordering of healthcare spending per capita. Don Berwick recently outlined a chartered agenda for health services and policy. His top two topics aligned with the ‘triple aim’ framework of better health for individuals, lower per capita costs (better value), and better health for populations alongside better ways to involve doctors in change and creating transitional business models for hospitals.^13^

HSPR has emerged as a mature field of study tied to evaluating and supporting implementation of health services improvements for both individuals and the health of the population. We suggest that HSPR researchers, funders and policy-makers consider a simple proposal. We need leadership to advance an international forum for cooperative exchange among voluntary, OECD national research agencies to track and advance common nomenclature, definition and categorization of HSPR in different national contexts. This is particularly important with healthcare spending consuming ever larger fractions of gross domestic product (GDP) and with questions of value looming. Such a forum could calculate the spend and work to track reform impacts arising from this work. There does appear to be some work emerging throughout the European Commission on health systems, which is pursuing some common effort on health services definitions.^20^ Policy-makers and research funding agencies can learn from comparative effects of HSPR investments across their health systems. While research is only one input to policy, such a collaborative effort of HSPR funders, research leaders and policy-makers would help our respective jurisdictions begin to untangle the complex science of attribution linking HSPR investment strategies and health system improvements.

As we work to untangle some of the attribution of HSPR investments in health outcomes, a case could be made for more precise earmarking and targeting of HSPR funds as a fraction of health spending, where the causal science might suggest. There is a well-respected UK precedent for earmarking 1.5% of health spending for research as was done following the prescient Culyer Report on Supporting Research and Development in the NHS more than two decades ago.^21^ A better alignment of HSPR definitions, a robust earmarking of spending for those effective HSPR investments which are instrumental in yielding better health outcomes, along with healthy competition on HSPR across nations can only lead to better performance in our health systems.

## Ethical issues

Not applicable.

## Competing interests

Authors declare that they have no competing interests.

## Authors’ contributions

Both authors collaborated on the concept and design, acquisition of data, analysis and interpretation of data, drafting of the manuscript, critical revisions of the manuscript for important intellectual content, and statistical analysis. Authors have no sources of funding to declare for this work. Both authors also provided the administrative, technical, and material support for this work. VT was supervised by TS as a part of a Senior Fellows learning activity at the Institute of Health Policy Management and Evaluation at the University of Toronto, Toronto, ON, Canada.

## Endnotes


[1] The conversion rate for currency was applied for all monetary values from CDN to USD and British Pounds to USD. The Canadian FOREX conversion calculator was used for this task: http://www.canadianforex.ca/currency-converter.

[2] For the United States, Denis and Associates provided estimates of public spending in HSPR. For Canada, Sullivan & Associates verified estimates for public spending in HSPR. For the United Kingdom, Dr. James Carter, the Evaluation Officer at the Medical Research Council verified the numbers within the UK research and analysis document.

[3] In all three countries, HSPR enjoys varying levels of support from charities, hospital associated foundations, and even direct spending on consulting services. We focused on government only spend on HSPR since it is most easily verifiable and the other charitable and private sources of HSPR funds, while not trivial, are not reliably captured or aggregated in comparable ways.

[4] http://cahs-acss.ca/wp-content/uploads/2015/07/ROI_FullReport.pdf.

